
JOURNAL INFORMATION
==============================
NLM Title Abbreviation: Med Chir Rev
NLM Journal ID: 0034617

The Medico-Chirurgical Review

Publisher: Biomedical Journal Digitization Project

ARTICLE INFORMATION
==============================
PMCID: PMC4966249
PMID: 29917621
Article version: 1
Subjects: Reviews, Diseases Produced by Malaria

The Morbid Anatomy of the Bowels, Liver, and Stomach, Illustrated by a Series of Plates, and a Summary of the Symptoms of the Acute and Chronic Affections of the Above-Named Organs

Print publication date: 1828 Oct 1
Volume: 9
Issue: 18
First page: 347
Last page: 358
1828 Aug
License: This work is free of known copyright restrictions. For more information, please see PMC Back Issue Digitization.
License URL: https://creativecommons.org/publicdomain/mark/1.0/

==============================

In our next article we shall take up the important subject of Chronic Hepatitis, a disease now imported annually into this country, so as to make it a matter of anxious investigation with every medical practitioner. An accurate acquaintance with the acute forms of Indian Hepatitis, however, is indispensable to the study of the chronic forms, and for this purpose we have laid before the English reader a very comprehensive analysis of the facts and observations recorded in the great work before us.

---

### VIII.

*The Morbid Anatomy of the Bowels, Liver, and Stomach, Illustrated by a series of Plates, from Drawings after Nature, with explanatory Letter-press, and a Summary of the Symptoms of the Acute and Chronic Affections of the above-named Organs.* By John Armstrong, M.D., Lecturer, &c. Fasciculus I. and II. 4to. pp. 70. with ten coloured plates. Baldwin. June & July, 1828.

[Art. I. Fasciculus 1.]

Dr. Armstrong's works on Typhus, &c. which appeared some ten or twelve years ago, procured for their author a reception and patronage from the professional and general public, such as have never before been witnessed or experienced in the annals of medicine. From being a successful author, Dr. A. became, in a very short time, one of the most successful candidates for private practice, in this great metropolis, whither he came a total stranger—and, from a successful career in practice, he turned his attention to medical tuition, where he quickly drew a larger class of students than any other physician in London. Some people may say this was all good fortune—or chance. But we have an idea, that to take such a lead in three different and difficult walks of the profession—writing, practising, and teaching—requires something more than the chapter of accidents, the patronage of friends, or the caprice of fashion.* A single glance over the two fasciculi before us convinces us, had we no other rea-

* Whenever a medical man arrives at any distinction by his writings or his practice, three fourths of his *contiguous* brethren divide into two sects—one, (a very small party comparatively) extolling him to the skies, and seeing in him nothing but excellence—the other (a tolerably numerous band) depreciating the fortunate candidate, running down every thing that comes from his tongue or pen, and ascribing all his success to the indiscrimination of mankind, and the blindness of Fortune. Happily there is a third and very influential personage, called the public, who, being far removed from the sphere of private and personal feelings, forms a tolerably correct estimate of men and things in the long run. It was but the other day we heard the following fragments of a dialogue between two medical gentlemen who met in Regent Street:—

A. "Have you seen Dr. Armstrong's Plates?"

B. "Yes."

A. "Were there ever such *daubs* offered to the medical profession? The two fasciculi are a disgrace to any physician!"

Now if we might be allowed to judge, we would say, that the plates are very superior, but that we cannot vouch quite so much for the letter-press; though the meagreness of the latter may be accounted for by the circumstance that Dr. A. is preparing a larger work on the same subjects.

sons for conviction, that Dr. Armstrong has effected a new æra in medical tuition. The introduction of highly finished drawings and models of natural and diseased structures, into purely medical lectures, has never been put into execution on any large scale, till Dr. Armstrong fortunately struck out the path—and the consequences are sufficiently obvious.* These drawings and models are infinitely superior to anatomical preparations, which never can retain any great verisimilitude to the structures during life. By these auxiliaries to oral instruction, the eye is made to assist the ear, not only in the conception, but in the retention of pathological knowledge. Dr. Armstrong is entitled to great praise for the introduction of these aids to medical instruction, because his example *must* be followed by his cotemporaries. The plates in this work do honour to this country, and have never been equalled for beauty of execution and accuracy of representation. This is saying a great deal, after the plates which have appeared from Mr. Annesley and Dr. Bright; but we believe we are perfectly safe in the opinion which we have here given. We must now proceed to the letter-press.

Dr. A. observes, in his preface, that two of the most prominent causes which have tended to retard the progress of morbid anatomy in this country, are—"prejudice on the part of the public, and something very like indifference on the part of the profession." He thinks it easy to account for the former, "on those inherent principles of our nature, which lead us to regard as inviolate the relics with which so many endearing recollections of life are associated."

"Yet it would be difficult to assign any very satisfactory reason for the professional indifference, once so extensively diffused, unless it owed its origin to that scholastic system of education, which directed the mind to nosological technicalities and metaphysical abstractions, rather than to the particular details and general inductions of pathological anatomy." *Pref.*

We fear there is some truth in this severe reflexion on medical education!

It is not Dr. A.'s design to delineate all the morbid appearances connected with the organs mentioned in the title-page, but to select those which are the most important, and which have been examined by his own hands and eyes. The annexed symptoms also are drawn from personal observation at the bed-side of sickness.

Morbid anatomy, Dr. A observes, appears a vast field at first sight; but if, for instance, we take the acknowledged products of inflammation, as seated in this or that texture, and to them add tubercle, scirrhus, fungus, and melanosis, we have at once a bird's-eye view of the most important changes which occur in the solids. But the fluids are directly or indirectly concerned in almost all morbid conditions of parts, and these must be taken into consideration. From some good general observations on the changes that take place in the quantity, quality, velocity, distribution, secretions, &c. of the blood, we shall select the following extract.

"Again, in some cases of fully developed typhous fever, where the tongue was glazed, dry, and brown, and the lips and cheeks of a dusky or purple hue, the blood drawn from a branch of the temporal artery had the venous colour. The circulation of such blood within the arteries is connected with many of the most conspicuous and curious phenomena of the advanced stage of genuine typhus. The cause of this remarkable change can be shown, by dissection, to depend upon a specific Bronchitis, the mucous texture of the bronchial tubes being loaded by dark blood, and be-

* We ought to except Dr. Thompson, of Edinburgh.—*Ed.*

smeared by a copious and tenacious secretion. The experiments of Dr. Edwards have proved, that two changes take place in the blood during respiration, namely, that carbonic acid gas is secreted directly from the blood, and that the oxygen disappears, not having combined, as was formerly supposed, with carbon, to form, thus indirectly, the carbonic acid. Now in the bronchial affection attendant on typhus, one or both of these processes are defectively performed, for the venous blood is returned, imperfectly changed, to the left chamber of the heart, and the circulation of this dark blood in the arterial system is, next to the original taint of the remote occasion, one of the most remarkable changes in the blood in cases of confirmed typhus. Browne Langrish, a century ago, inferred from experiments that the proximate principles both of the blood and of the urine were changed in fever. But with respect to typhus this is not always the case in the beginning, except where Acute Inflammation takes place in a serous membrane, with high excitement, or where an extreme Bronchitis exists, with an imperfectly developed excitement; and then the buffy coat is shewn on the blood drawn in the first case, while in the last, the blood usually remaims fluid, and the relative proportion between the cruor, fibrine, and albumen often undergo remarkable alterations, apparently from the influence of the Bronchitis, which, by impeding the offices of respiration, materially affects the assimilation of the blood. The proximate principles, too, are changed towards the middle and advanced stages of typhus, rather by the defective digestion attendant on the mucous irritation of the alimentary canal, and the defective assimilation attendant on the bronchial affection, than from the direct agency of the specific taint from which the disorder originally arose; for I have found similar changes in the proximate principles of the blood both in Acute and Chronic disorders, which sprung from remote occasions of a Common kind, and which could not communicate any contamination at once to the blood. As far as the remote occasions and ultimate effects are concerned, fevers might be advantageously divided into Common and Specific; the Common proceeding from the ordinary agents of nature, such as heat and cold, used in the popular sense, and the Specific arising from peculiar agents, such as putrid matter, and the human contagions. Even in Common fevers, it would be easy to prove that the blood undergoes sometimes very striking alterations, but this is more especially the case in Specific fevers, the peculiar occasions operating on the blood, and that again, in its turn, influencing the condition of certain solid textures. For example, in puncture from dissection three effects are produced. The first a mere local pustule, without any remote disturbance; the second a considerable topical inflammation, which produces fever, on the ordinary principle of irritation, of a common character from beginning to end; but there is a third and more formidable effect, namely, an absorption of putrid matter, in general from a slight wound, which, tainting the blood, produces true typhous fever, usually of the continued type. Now if putrid matter be so introduced into the blood as to give rise to a distinctly marked fever of this Specific sort, the brain and its membranes, the bronchial lining and that of the small intestines are invariably, so far as my dissections have extended, affected by the appearances of Increased Determination or Inflammation, when the disorder runs its definite course of about three weeks; and as these appearances are found in the male and female, in the young and old, whatever may have been their temperaments or habits, so it is reasonable to conclude, that the contamination of the blood operated specifically on the forementioned textures. If it be contended, that the capillary injection of all the above-named parts is the effect of the continued excitement, the objection admits of a ready answer; since in Common fever, in Acute rheumatism for instance, when the excitement is often higher, and remains longer, such effects are not uniformly displayed. In like manner, the contagions of small pox, measles, and scarlatina first operate on the blood, and that fluid being thereby changed, the solids are specifically affected, especially the skin, and mucous membrane of the air passages; and these affections, too, if left to themselves, and even often in despite of medical applications, have a determinate course, the blood, apparently, like the water of the Thames, requiring a certain time for its purification, which it effects, perhaps, by throwing off the effete and superfluous matters, through the secretions and excretions." 10.

Believing, as we do, that the progress of knowledge tends more to reveal our weakness than increase our strength in therapeutics, we are not so sanguine as Dr. A. in the following aspiration :—"The time may come, and probably will come, when disorders, whether acute or chronic, which arise from specific occasions, (as the eruptive diseases,) will be directly removed by remedies acting through the blood, and thus the structure of the solids saved from that indirect derangement, so frequent under the existing and imperfect state of our art." We cannot follow Dr. Armstrong through a series of elementary observations on the state of the blood, and of the various secretions in disease. They are well worthy of attention. The following passage bears on a disputed point of pathology, and will be read with interest.

"The tubercle has been supposed by an able and ingenious author, Dr. Baron, to commence as a vesicle, and to be nothing more or less than an hydatid, if I rightly comprehend his meaning. But I have purposely noted a great variety of cases with much care, and found that the vesicular appearance of the Tubercle is simply an accidental occurrence, dependent on the texture of the part in which it is placed. Thus, for example, in their origin, Tubercles may have the vesicular appearance in the lungs; but these, if minutely examined, will be found to be the extremities of the bronchial tubes, or air cells, into which the peculiar deposit, constituting tubercle, often takes place. Frequently I have examined, under a strong light, Tubercles on the serous membranes, and have never yet found them, strictly speaking, vesicles there, though the tubercular points, in many cases, have been extremely minute. It appears to me, that Tubercles are secretions, from the ultimate ramifications of the arteries, called the exhalents; for I have preparations in which they seem to hang from the arterial capillaries like bunches of grapes from the shoot of a vine-branch.* Tubercle generally begins in a semi-opaque point, it becomes wholly opaque, and is then often of various sizes, but most frequently of a millet or mustard-seed. It does not, so far as I have tried, admit of injection at this or any other subsequent stage. It may remain latent for a long time in the primitive semi-opaque, or secondary wholly opaque condition; but it in general undergoes a third change, increasing in size, it may be, to that of a small pea, or several Tubercles running together may form a much larger mass. Having been once progressive in the last-mentioned mode, they generally undergo a fourth change, soften in the centre, and at last are resolved throughout their whole substance by a process apparently analogous to that of suppuration. Sometimes Tubercles are enclosed, like a kernel, in a thin or dense capsule, particularly when they form in glands; and sometimes the cavities occasioned by their solution are surrounded by a false membrane of effused fibrine; but, in the lungs, the mere parenchyma often forms the walls or boundaries of a vomica, or excavation. The number and the increase of the size of Tubercles frequently create irritation in their vicinity, so that a consequent Inflammation of the surrounding texture is not an uncommon circumstance, as may be daily witnessed in the dissection of bodies dead of phthisis pulmonalis. If, as above stated, Tubercle be a secretion from the arterial terminations, it follows that it is but the effect of some preceding change in the solids or fluids, or in both these. Is it one of the products of inflammation, or is it a peculiar deposit, the immediate pathological causes of which are as yet unknown? Certainly, if the matter of tubercle be examined, it more sensibly resembles the fibrine or albumen of the blood than any other material. But it may be said that the fibrine or albumen of the blood, when effused, during disease, is an organizable substance, and that tubercle, not admitting of injection, is not apparently endowed with that property. It might, however, be answered, that effused fibrine or albumen

* "To show tubercles in this state, it is requisite for the part to be macerated in simple water, exposed to the sun, without a cover, till putrefaction take place, and then it should be washed under a gentle stream, such as that from a cock in a barrel, daily, until the parenchyma of the lung be completely separated."

is not always organizable, as we perceive in weak subjects affected by pericarditis and pleuritis, where loose lymph often exists abundantly, without any adhesions. Moreover, it might be urged, that our incapacity to inject Tubercle is not a perfectly conclusive proof of its want of organization, the vessels being, possibly, too minute to admit the artificial fluid, and the growth and solution of the Tubercle contribute to show that either it, or a delicate membrane which so often invests it, may be endowed with a low degree of vitality, by which it increases till it reach a certain extent, and then passes into decomposition, like many other productions of nature. Against the idea, however, of Tubercle being simply the effect of Inflammation many facts might be adduced, but one may suffice. In many instances, where tubercular points are scattered over the pleura or peritoneum, the serous membrane is transparent up to these points, and only becomes reddened or opaque when the tubercle has become progressive and enlarged, so as to act as a local irritant. Yet it seems probable, that Tubercle is connected with an effusion of the fibrinous modification, though, according to my observations, that effusion is not necessarily connected with Inflammation. Certainly, Tubercle and Inflammation are often co-existent, and so are the hydatid and Tubercle occasionally, but co-existence does not imply a direct dependence or relation. The tendency to Tubercle, like that of other extraneous formations, is strikingly hereditary; and indeed the miliary Tubercle, perhaps the germ, as Laennec supposes of all the rest, has been found in great numbers in the lungs of still-born children; but in every case of this nature which has fallen under my own observation, the mother was tubercular, or tabid, during the period of gestation. Nevertheless, it seems to me quite certain, that Tubercles may be, and frequently are, generated, *de novo*, in the human body and in the lower animals. In almost every instance, when I have traced their development after birth, two circumstances have preceded, namely, a previous condition of debility, accompanied by some degree of attenuation; and, secondly, preternatural paleness of the skin, with co-existent signs of irritation on some portion of the internal mucous texture; and to these changes, whether induced by evacuations, mercury, protracted disorders, bad diet, anxiety, night watching, sedentary habits within doors, or any other enervating circumstance, the operation of a low or variable temperature has generally been superadded, as an exciting occasion to the prior state or predisposition. This doctrine, in a preventive view, is highly important, and it shall be confirmed in the subsequent pages by many facts and arguments, which will prove, not only that Tubercle in general is the ultimate result of an antecedent change in the fluids and solids, but that its development may be prevented, in many cases, by preserving the general strength entire, and enabling the surface, through cool ablutions, and daily and much exercise in the open air, to sustain the shocks of our fluctuating climate; for Tubercles are, in most instances, only produced in the internal organs of an ill-conditioned subject, as vermin are said to be formed upon the skin, when it is kept uncleanly, and when the body is shut out from the wholesome influence of light and air. The existence of tubercles in the lungs of still-born children cannot be held as a valid objection to the view here taken, since the fœtus was formed within a sickly mother, whose blood conveyed to it, perhaps, the materials of contamination, nay, its body and imperfect health might be regarded merely as integrant parts of the mother herself. Upon inquiry, it will be found that those persons who are made the most delicate by domestication and other debilitating causes, are the most prone to consumption, and the same assertion is applicable to the lower animals; whereas, those persons, such as gipsies, hawkers, and the like, who are continually in the open air, provided they be temperate, are the least liable to tubercles; and it would be easy to prove that the same doctrine is verified by a review of the habits of the lower animals, more especially of the sheep and horse." 18.

Dr. A. has never been able to trace scirrhus to the capillary exhalents, though he thinks it probable that it proceeds from those about the part affected. It is curious that scirrhus and tubercle very rarely co-exist in the same individual. Our author has seen but one instance of their cotemporary existence.

"If what many pathologists have termed the scirrhous liver of a confirmed drunkard be properly macerated, we shall often find that the interstitial deposition being washed away, the arteries, even the minute ones, are thickened and almost cartilaginous, seemingly from inflammation, appearing not very dissimilar to the cancelli of some bones, or to the blanched interlayings of net-work. The interstitial deposition sometimes appears to exist in patches, and sometimes pervades the whole liver; it is of considerable firmness, and commonly of a greyish-white colour; and though in a recent state it cannot be squeezed out, yet by long maceration first, and pressure between the fingers next, it can always be separated, so as to leave the true character of what has been designated by the term Cancellated Induration. In one case, a solid fibro-cartilaginous patch existed in the liver corresponding to the ordinary Scirrhus, but a little above this patch the Cancellated Induration was apparent, and seemed to be lost in the other. Has Scirrhus any connection with chronic Inflammation of the capillary or larger vessels of the affected part? Has it any connection with the veins of the part, into the calibers of which the scirrhous matter is sometimes effused? More minute attention than has yet been paid to the subject would be requisite to authorise any one to give an unqualified answer to these questions." 20.

Simple induration is very common, and arises from the effusion and subsequent organization of lymph in the parenchyma of any organ, as, for instance, in hepatized lungs. Softening, on the other hand, is also a product of inflammation. Scirrhus and fungus encephaloides are frequently found co-existent, though they are evidently very different morbid growths. The latter is generally enveloped in a cyst, but is sometimes found dispersed among the different textures of the body. In respect to melanosis, Dr. A. considers it a "secretion sometimes occurring in textures otherwise apparently natural—sometimes in those chronically inflamed—and sometimes co-existent with either scirrhus or fungus." The author is evidently inclined, therefore, to view the disease as one of the fluids rather than of the solids.

"As far as my recollection serves me at present, every case of Melanosis which I have witnessed was accompanied by more or less of chronic bronchitis; but it would require the care of other men to determine whether this conjunction be constant, or merely occasional; for no error has been so prejudicial in medical philosophy as universal conclusions, drawn from a limited number of particulars. It is clear, however, that bronchitis, simply of itself, is not sufficient to produce Melanosis, numerous cases of the former occurring, in which, on examination after death, no trace of the latter is any where discernible. But, on the other hand, is it at all probable, that bronchitis may be *one* of the precursory or concurring causes of Melanosis? Is it possible that, by changing the whole mass of blood into a more venous character, bronchitis may favour the dark and peculiar secretion, respecting the composition of which chemists have differed?" 25.

The first fasciculus of the work before us is dedicated to the STOMACH, the principal morbid affections of which are classed under the heads—"increased determination—inflammation—scirrhus—and fungus encephaloides." It is remarkable that Dr. A. should not have included NEURALGIA of the stomach among its principal affections, since, if the frequency of its occurrence, and the distress occasioned by it be of any importance, the disease ought to have occupied the first rank, instead of being put out of the ranks almost entirely.*

* There are a few lines respecting the gastric neuralgia of young women who starve themselves in order to get lean, but very little or nothing on the great class of neuralgiæ connected with indigestion.

In respect to inflammatory affections of the stomach, Dr. A. appears desirous of steering a middle course between the Continental and British pathologists.

“ Some pathologists on the Continent seem to think, that Inflammation of the Stomach very frequently occurs, especially in the mucous texture ; whilst some in this country conceive, that, though this viscus be greatly abused by our artificial and luxurious habits, it is nevertheless, but seldom the seat of actual Inflammation. The truth appears to lie between the extremes of these opinions ; for while I am ready to admit, that Increased Determination has often been mistaken for Inflammation of the mucous texture of the stomach, yet I am equally certain, that Inflammation is not so very uncommon a circumstance, oftener, indeed, assuming the chronic than the acute character. Increased Determination is not accompanied by the combined symptoms and progressive effects of Inflammation during life ; and though, after death, the mucous texture of the stomach be found red by a preternatural injection of the capillary vessels, yet none of the genuine products of Inflammation are present, such as effusion, softening, thickening, or so forth, and it may thus be easily discriminated from the latter.

“ When the body is examined shortly after death, the vestiges of acute and chronic Inflammation of the serous and mucous membrane of the stomach are generally very distinct. In acute Inflammation, the capillary vessels, but more particularly the venous ones, are much injected by red blood; and there are, besides, some of the palpable products of Inflammation connected with effusion. These circumstances, being conjoined, are indisputable evidences that Inflammation had existed. The injection of the serous membrane is most frequently arborescent, the minute arteries and veins shooting, as it were, like the branches of a tree, in different directions, or, what may perhaps be a more apt comparison, winding across, like the vessels seen in a leaf when held between the eye and the light. Sometimes the redness, in part, is irregularly dotted, smaller and larger points appearing here and there, as if partial exudations of blood had taken place; yet, if these points be accurately examined, they will be mostly found nothing but engorged capillaries, in all likelihood the exhalents themselves, which, being still more extremely loaded, probably admit of that actual exudation that does occasionally occur. The serous membrane over the site of the Inflammation loses its natural transparency and becomes more or less opaque. Another circumstance remarkably characterizes acute Inflammation of the serous membrane, namely, that it is much more easily separable than natural. Indeed, it may be generally stripped off by the fingers, just as the rind is peeled from a ripe orange. Fibrine, pus, or some effusion of an intermediate character, is usually seen on its free surface, while serum is found in the vicinity, commonly discoloured by an admixture of one of the forementioned products. In most of such instances, a large quantity of gas is generated within the stomach, evinced by the extreme distension of that organ even during life.

“ One of the most striking differences between acute and chronic Inflammation of the serous membrane, but more particularly that of the stomach and bowels, is the greater injection of the larger as well as small branches of the veins in the chronic, by which a darker colour is given to the part. Having seen this condition of the veins an almost constant attendant, I have inferred that these vessels are more intimately concerned in the phenomena of Inflammation, especially when chronic, than has been allowed in our reasonings on the subject. Not only is the opacity more evident in the chronic Inflammation of this texture, but the thickening more considerable and conspicuous. The late most able and excellent Laennec supposed, that serous membranes were not really thickened by Inflammation, but that the effusion of fibrine becoming organized on their free surfaces gave rise to the deception. Though such an apparent thickening be not uncommon, nevertheless it is not always thus produced. Bichat supposed that the serous membrane was composed of one layer; but if we macerate it for about ten or twelve weeks, in nearly equal portions of vinegar and water, we may divide it, by nice management, into two or even more laminæ, as shown by specimens in my possession. The laminæ are knit together, by cellular membrane, into which the effusion of fibrine sometimes takes place so copiously, that the substance of the serous membrane is really thickened. Where fibrine has been interstitially poured out in this way, an increase of density is the necessary consequence; but when a more albuminous, gelatinous, or serous fluid has been principally effused, which does not admit of subsequent organization, the serous membrane is often pulpy, as if from maceration, an appearance most frequent in lax habits. Softening is a common, but not a constant attendant of chronic inflammation, as may be perceived, where adhesions exist between the serous membrane of the stomach and that of the liver, or between one portion of this texture and another covering the coils of the intestines. Ulceration of the serous membrane of the stomach is a rare occurrence from common inflammation, at least, I have only met with it occasionally; in the one case, occurring originally in that structure; in the other, reaching it through an extension of disease, from the mucous lining of that viscus." 29.

When the mucous membrane, on the other hand, is acutely inflamed, the remaining redness is intense, and generally diffused as if by a brush over the greater portion of its surface—sometimes circumscribed in broad stripes or patches gradually shaded or abruptly breaking off, with apparently sound intervening portions.

"At first sight, the acute inflammation of this texture has a close resemblance to painted velvet, it appears so red and raised; but on a closer inspection, the redness will be found to be partly of the arborescent, and partly of the dotted form. The combinations can be distinctly seen, if the inflamed portion of mucous membrane be spread upon the clean pane of a window, the redness then having at once a ramified and freckled appearance. Thickening, puffiness, softening, and easy separation, usually mark inflammation of this membrane;* but they are more manifest in that modification of inflammation which might be justly called sub-acute, and which stands between the acute and chronic, being less urgent, and more protracted than the former, though of much shorter duration than the latter. These changes, too, accompany chronic inflammation of the mucous membrane, which then generally has a gelatinous appearance, blended with an irregularly liny, and almost freckled state, the first, from the partial softening of that texture, and the second, from the ramification of the minute subjacent vessels; while, at the same time, the mucous follicles appear either prominent, like papillæ, or are actually ulcerated, and the veins in the serous membrane are injected and enlarged. The existence of these follicles makes a considerable difference in the pathology of inflammation of the mucous membrane compared to that of the serous, as will be amply elucidated in the sequel." 30.

It is not the least of the anomalies presented in the science of medicine, to find the most opposite effects resulting from the same cause. Softening is equally the product of inflammation as induration. Phlogosis of the stomach will often leave a milky whiteness as well as an intense redness—thickening and attenuation of the coats depend on the same cause! These anomalies, in many instances at least, appear to our author to be connected with the state of the arteries or of the veins of the tissue, according as one or other set of vessels is principally concerned in the phlogistic process. This seems rather inclining to the hypothetical.

* "By long maceration, the mucous texture of the bowels may be shown to consist of three laminæ, which almost appear like cobwebs, and which are seemingly united by cellular texture, resembling so much fine intervening lace-work. It is this cellular structure which is the main seat, in the different organs of the body, of most of our diseases."

### SOME NOTICE OF THE PLATES.

The first plate is that of the stomach, representing the red suffusion and arborescent injection of acute inflammation of the serous membrane, with a deposition of fibrine on its surface, and some between its laminæ. It is one of the best plates in the work.

The second plate was copied from a stomach, in which the inflammation commenced under a sub-acute form, and terminated in three weeks. The mucous texture was found highly injected, pulpy, and thick, with some ecchymosed spots.

The third plate shews attenuation, with solution of some of the textures of the stomach, from inflammation. The fourth is perforation of the organ in a child; and the fifth shews the thickened and pulpy state of the stomach from muco-gastritis and sero-gastritis. All these are done on stone, and the artists are entitled to great praise. Mr. Cocks, who made the drawings, is a gentleman of great merit. The colouring is much too high in most of the plates—a common fault in all pathological representations.

### SYMPTOMS.

We must now proceed to give some account of our author's symptomatology, which is laconic, but evidently copied from nature.

1. *Acute Inflammation of the Serous Membrane of the Stomach.* The following is Dr. A.'s symptomatology of this important phlogosis.

"If the serous membrane be alone acutely inflamed, there is an urgent pain in the region of the stomach, increased considerably by pressure there, and even by a deep inspiration. The breathing is hurried and anxious, the skin hot, the pulse very quick, and remarkably small, but for some time it will be found harder or more incompressible than in health, only becoming really weak and soft towards the close. The tongue is covered by a whitish fur, and the stomach is very flatulent and irritable throughout, nausea, retching or vomiting being present, especially when any food or medicine is given.

"There are two distinct stages in all acute serous inflammations—one of excitement, and another of collapse. In the stage of excitement the heat of the skin is higher than natural, the pulse, however small, more resisting than natural, and the respiration, though rapid, is not embarrassed; whereas, in the stage of collapse, the heat falls first on the extremities, and then on the trunk, the pulse grows soft and weak, and generally quicker than before, while the respiration is carried on feebly. These two stages are usually well marked in acute inflammation of the serous membrane of the stomach, the vomiting becoming more urgent, the skin damp as well as cold, and the face sunk in the last stage, or that of collapse." 41.

2. *Subacute Sero-Gastritis.* Although this differs only in degree from the former, pathologically speaking—yet this difference in degree is of considerable importance, and justifies the distinction. In this grade the fever is lower, and the local disturbance of the part affected is less—the progress is slower, in the proportion of two or three weeks to a few days. Vomiting is often absent in the subacute form, there being more frequently only a loathing of food, with occasional nausea, till towards the conclusion, when vomiting supervenes.

*Chronic Sero-Gastritis.* "In Chronic Inflammation of the Serous membrane of the Stomach, fever is either wholly absent, or, if present, it has a slow consuming character. It is not, so far as my dissections have gone, a common circumstance to find Chronic Inflammation confined to the Serous membrane of the Stomach alone, it generally being complicated with Chronic Inflammation of the Serous membrane of the Bowels, or of the Liver. Chronic Inflammation of the Serous membrane of the Stomach is at all times obscurely denoted, but the forementioned conjunction tends to make the diagnosis more difficult. This form of Inflammation, however, may mostly be detected, by the symptoms having a permanent seat and character, and by the effects of the disorder being increasingly marked on the frame at large. There is more or less pain in the epigastric region, aggravated by moderate pressure, and accompanied by a sense of distention and confinement, particularly after any thing like a full meal. There is constant uneasiness about the stomach. It may be at times obscure, but is very liable to be increased by whatever offends that organ, which is then always more flatulent and irritable than natural. The flesh wastes, the skin acquires a sickly hue, the mouth is dry or clammy, the tongue is covered with a whitish fur in the centre, and is not only pale about the tip and edges, but often appears as if it were broader than before the attack.

"Pathological anatomy points out the propriety of discriminating Serous from Mucous Inflammation, and we might, therefore, call the above described modifications, Sero-Gastritis, and when that condition affects the mucous texture, we might call it Muco-Gastritis, the symptoms of which are next to be enumerated."

4. *Acute and Subacute Muco-Gastritis.* In this affection, the pain is of a more burning kind than in the acute sero-gastritis, and the desire for cold drink more insatiate. The pulse is softer—the fever less ardent—" the tongue is pale at the point and sides in acute *sero-gastritis*—but in muco-gastritis the tongue is vividly red at the point and some way round the margins, nay, often thus coloured over a considerable portion of its surface."

"These are the symptoms *proper* to each of the above varieties of Acute Inflammation, a concentration of heat about the Epigastrium, irritability of the stomach, and anxious respiration being *common* to both. The same remarks are applicable to Sub-Acute Sero-Gastritis and Sub-Acute Muco-Gastritis when contrasted; but it must be constantly remembered, that there is less fever and less local disturbance in the Sub-Acute than in the Acute Inflammation, while the former is of longer duration than the latter, these being the three essential points in which they differ from each other." 43.

5. *Chronic Muco-Gastritis.* This is a far more common disease than the acute or subacute forms.

"Chronic Muco-Gastritis is attended, generally, by a vermilion tint of the tongue, at the tip and edges, while the papillæ, for the most part, are red, and also raised, somewhat like the points upon a strawberry.* But the most certain sign is pain or uneasiness uniformly after meals, which increases as the disorder goes on, and which is at length accompanied by general wasting, and an acceleration of the pulse, with pallidity of the skin and slow fever. The temper, too, is more easily ruffled than natural, or the spirits depressed. If a doubt exist as to the nature of the disorder, it may generally be removed by the exhibition of a diffusible stimulus, which always increases the uneasiness in the Stomach, if Chronic Muco-Gastritis be present, so certainly does the system, in this case, resist or indicate the impropriety of the stimulant treatment. Now that this treatment is so prevalent, from the remains of a vague and erroneous philosophy respecting dyspepsia, the young practitioner would do well to recollect the test already named, which may be safely relied upon in almost all dubious cases; and, on the other hand, as some modern pathologists press the doctrine of Inflammation beyond its legitimate bearing, it is

* "Chronic Muco-Gastritis and Chronic Bronchitis are often conjoined, and then the papillæ of the Tongue have a purplish cast."

equally needful to be guarded on that point, lest evacuations should be used where stimulants are demanded.

"In fact, there is a painful affection of the Stomach, which is not inflammatory, and which is relieved by stimulants. It mostly attacks those who have suffered much from anxiety, or who have been considerably fatigued or exhausted. But it may arise in robust persons, from any article of diet very indigestible, or from a complicated meal. In this affection the pain is usually severe, without the least degree of fever, and it is accompanied by feelings of distention, weight, and fermentation. It is in general soon allayed by drinking hot water, or by the exhibition of laudanum, but the most rapidly and effectually by that of pure brandy. This kind of pain has long been distinguished in popular language by the term Spasm of the Stomach, or of Windy Colic when it exists in the bowels, and the value of alcohol is well known to the vulgar in such cases." 44.

Our author observes that organic diseases of the heart are not unfrequently associated with chronic inflammation of the mucous coat of the stomach, especially towards their termination. In such instances, preternatural sensibility of the stomach, with nausea, retching, and vomiting, together with the crimson tongue, mark the nature of the ventricular disorder.

"If ulceration exist on the Mucous surface of the Stomach to any extent, patches or points of pus may now and then be discovered in the matter vomited, and these are mostly mixed with a glairy tough mucus, or streaked with blood. In the advanced stage of Chronic Muco-Gastritis, patients sometimes vomit considerably more fluid than had been drunk, and this is most liable to happen in those cases where the inner coat of the Stomach is blanched and soft, which occasionally occurs in truly inflammatory cases." 45.

In respect to dissolution and perforation of the stomach, Dr. A. places little or no belief in the solvent powers of the gastric juice. In all the instances of perforation which he has seen, "the most unequivocal signs of disease existed before death."

"In those cases, which occurred in adults, a sudden and severe pain arose, with vomiting, as if the patient had taken an acrid poison, and the fatal stage of sinking took place within forty-eight hours from the attack; but the infant, whose Stomach is represented in the fourth Plate of the first Fasciculus, lived about seven days after the violent seizure, having been previously weaned, and weakly, from the history of its mother. The dissolution of the Stomach was announced by sudden and severe fits of crying, attended by a distressing sickness, and retraction of the lower limbs towards the abdomen. The epigastrium was hot—the integuments of the belly hard—the pulse quick—and the respiration anxious. Diarrhœa supervened—the face gradually assumed the hippocratic character, and the extremities became cold. Such were the leading symptoms in the other cases which happened in my practice after weaning, all having been more protracted than those of the adults. It is not my intention to deny, that the Gastric juice may dissolve the Stomach now and then after death; but, in the preceding cases, disease certainly existed in that organ, and was apparently the cause of the dissolution. If it be asked, what was the nature of that disease, I answer, that I do not know. The Mucous membrane is sometimes attenuated and even destroyed by Inflammation, but occasionally similar changes take place from a process, seemingly, not inflammatory; and, as in the fore-mentioned cases of dissolution, the usual traces of ordinary inflammation were not present, it is not logical to refer the effect to that cause. It may be, however, that some change does take place in the mucous texture, or in the blood, by which the secretion is so altered as to act destructively upon the Stomach during life; but, as there is no end to conjecture where observations are too imperfect for legitimate deductions, I must leave this point of pathology to the consideration of succeeding inquirers." 47.

On perforation from chronic ulceration of the stomach we need not dwell. The symptoms of sudden peritonitis generally supervene, and death soon closes the scene. We have now finished the first fasciculus, and laid before our readers a very ample analysis and numerous extracts, by which they can judge for themselves. Did we indulge in rigid censure or indiscriminate praise we might be suspected, and justly so, of belonging to one of the parties described in the first page of this paper.

We shall proceed to other organic diseases of the stomach in our next article.

---

### IX.

*On difficult Cases of Parturition: and the Use of Ergot of Rye.* By W. Michell, Member of the Royal College of Surgeons. Octavo, pp. 128. Underwoods, 1828.

Among the blessings and curses attendant upon, or rather produced by, the progress of civilization, are crooked spines and lingering labours. Well! had it not been for the evil sent into this world, we should have been deprived of much of the good we possess. The diseases of the body have called forth the energies of the mind, and for every corporeal defect we have twenty different remedies. It is curious that the action of *specific* medicines (by which we mean medicinal substances that act *specifically*, or particularly on one organ or function) should have been more frequently discovered by the ignorant, or at least the empirical, than by the most enlightened and learned physicians. The reason, however, is not very difficult to assign. The ignorant look upon all medicinal agents as specifics, and consequently are always hunting after such agents. The learned have little faith in specifics, and are more employed in the investigation of structures, functions, and the general operations of drugs on the animal economy. We believe there is no substance better entitled to the title "specific" than the ergot of rye—a substance, the good properties of which were first appreciated by old women beyond the Atlantic. If we can credit the anticipations of the author whose work is before us, and who appears to be a sensible, a well-informed, and an experienced practitioner, this little abortive grain of rye will effect a greater revolution in medical practice than vaccination—and will go farther to bring back midwifery within the domain of its original professors, than all the orations of Sir Anthony Carlisle. The following extract will make our meaning intelligible.

"Every candid and honest practitioner will admit, that if we could discover any treatment which would effect delivery with safety to the mother and child, it would be preferable in other respects, and tend to the comfort of the Medical Practitioner, to resign the Midwifery branch again to the Female Attendant, except in some
